# Speech Perception in Older Adults: An Interplay of Hearing, Cognition, and Learning?

**DOI:** 10.3389/fpsyg.2022.816864

**Published:** 2022-02-17

**Authors:** Liat Shechter Shvartzman, Limor Lavie, Karen Banai

**Affiliations:** The Auditory Cognition Lab, Department of Communication Sciences and Disorders, University of Haifa, Haifa, Israel

**Keywords:** perceptual learning, degraded speech, hearing aids, aging, age-related hearing loss

## Abstract

Older adults with age-related hearing loss exhibit substantial individual differences in speech perception in adverse listening conditions. We propose that the ability to rapidly adapt to changes in the auditory environment (i.e., perceptual learning) is among the processes contributing to these individual differences, in addition to the cognitive and sensory processes that were explored in the past. Seventy older adults with age-related hearing loss participated in this study. We assessed the relative contribution of hearing acuity, cognitive factors (working memory, vocabulary, and selective attention), rapid perceptual learning of time-compressed speech, and hearing aid use to the perception of speech presented at a natural fast rate (fast speech), speech embedded in babble noise (speech in noise), and competing speech (dichotic listening). Speech perception was modeled as a function of the other variables. For fast speech, age [odds ratio (OR) = 0.79], hearing acuity (OR = 0.62), pre-learning (baseline) perception of time-compressed speech (OR = 1.47), and rapid perceptual learning (OR = 1.36) were all significant predictors. For speech in noise, only hearing and pre-learning perception of time-compressed speech were significant predictors (OR = 0.51 and OR = 1.53, respectively). Consistent with previous findings, the severity of hearing loss and auditory processing (as captured by pre-learning perception of time-compressed speech) was strong contributors to individual differences in fast speech and speech in noise perception. Furthermore, older adults with good rapid perceptual learning can use this capacity to partially offset the effects of age and hearing loss on the perception of speech presented at fast conversational rates. Our results highlight the potential contribution of dynamic processes to speech perception.

## Introduction

Aging is often accompanied by sensorineural hearing loss (presbycusis) and poor speech perception in daily listening environments (Committee on Hearing, Bioacoustics and Biomechanics (CHABA), 1988; Humes, 1996; Morrell et al., 1996; Pichora-Fuller, 1997; Gordon-Salant and Fitzgibbons, 2001; Dubno et al., 2008), especially under adverse listening conditions (e.g., in the presence of fast speech or competing noise; Pichora-Fuller and Singh, 2006; Schneider et al., 2010). There is tremendous variability in degraded speech perception among older adults. This variability is associated with sensory and cognitive factors (Committee on Hearing, Bioacoustics and Biomechanics (CHABA), 1988; Souza, 2016), as well as with individual differences in perceptual learning for speech (Karawani et al., 2017; Manheim et al., 2018; Rotman et al., 2020b). Hearing aids are the most common rehabilitation for speech perception difficulties in older adults with age-related hearing loss (Souza, 2016). However, like their non-hearing aid using peers, older adults who use hearing aids also vary widely on measures of speech perception. We hypothesize that the same factors that account for individual differences in degraded speech processing in adults with presbycusis are likely responsible for some of the variability in speech perception performance observed among hearing aid users. Therefore, the overall aim of the current study is to assess the relative contribution of sensory (i.e., hearing acuity) and cognitive factors (working memory, vocabulary, and selective attention), rapid perceptual learning, and the use of hearing aids to the identification of different types of degraded speech among older adults. We used three speech tasks—fast speech, speech in babble noise, and competing speech—which represent different challenges that can be encountered in daily listening situations, and which are known to pose difficulties for older adults with hearing loss (for review see Humes et al., 2012). The effects of cognitive factors, learning, and hearing aids might differ across these different conditions. Whereas the challenges associated with fast speech result from source degradation (speaking rapidly changes the temporal and spectral characteristics of speech, Koreman, 2006), the challenges associated with speech in babble noise and competing speech are associated with the listening environment (transmission degradation according to the terminology proposed by Mattys et al., 2012).

### Speech Perception in Age-related Hearing Loss

Age-related hearing loss is a primary contributor to speech perception difficulties in older adults (e.g., Humes and Roberts, 1990; Jerger et al., 1991; Humes, 2002). Individuals with age-related sensorineural hearing loss often require favorable signal to noise ratios to recognize speech due to elevated hearing thresholds (Killion, 1997). Reduced audibility (e.g., Gates and Mills, 2005), impaired temporal synchrony (e.g., Hopkins and Moore, 2007, 2011), and broadening of auditory filters (e.g., Peters and Moore, 1992; Leek and Summers, 1993) have also been suggested to account for the connection between age-related hearing loss and reduced perception of speech in noisy environments. Overall, it is estimated that these sensory factors account for 50–90% of individual differences in speech perception (for review, see Humes and Dubno, 2010).

When listening to connected speech (i.e., utterances longer than one word such as sentences or longer units of speech) older adults with age-related hearing loss often have perceptual difficulties with rapid speech rates (e.g., Tun, 1998; Gordon-Salant and Fitzgibbons, 2001; Wingfeld et al., 2006), in the presence of background noise (e.g., Pichora-Fuller et al., 1995; Helfer and Freyman, 2008) or in the presence of competing speech in dichotic listening situations (e.g., Jerger et al., 1994, 1995; Roup et al., 2006). However, auditory factors might be insufficient to explain individual differences in these situations because listeners with identical audiograms can have vastly different speech perception abilities (Luterman et al., 1966; Phillips et al., 2000; Schneider and Pichora-Fuller, 2001; Pichora-Fuller and Souza, 2003). Even when matched for audiological factors, older adults often find it more difficult than their young counterparts to perceive and comprehend speech in adverse listening situations (Gordon-Salant and Fitzgibbons, 1993; Needleman and Crandell, 1995).

The contribution of audiometric thresholds to speech perception tends to be larger in relatively easy conditions (e.g., identifying words in a quiet background) than in more challenging ones (e.g., with temporally distorted speech and speech in noise; Gordon-Salant and Fitzgibbons, 1993, 2001; Humes, 2007). Furthermore, whereas audiometric factors typically allow reasonably accurate predictions of speech in quiet, using auditory thresholds often leads to over estimation of performance of speech in noise (Dubno et al., 1984; Schum et al., 1991; Hargus and Gordon-Salant, 1995). Thus, once a task becomes more demanding, additional factors are needed to explain performance, as explained below.

### Cognitive Abilities and Speech Perception in Older Adults

Current models of speech recognition like the Ease of Language Understanding (ELU) model (Rönnberg et al., 2013) highlight the significance of cognitive factors for the processing of speech in ecological listening. The ELU model suggests that when the speech signal is degraded, for example, by competing noise or due to hearing loss, the automatic encoding of incoming speech may fail to match long-held representations within an individual’s mental lexicon. When such failure occurs, explicit processing of the signal becomes necessary to achieve speech understanding. This is done by utilization of previous experience and context, as well as recruitment of linguistic knowledge and more domain-general cognitive resources (e.g., working memory and attention) to support listening (Pichora-Fuller et al., 1995; Akeroyd, 2008). By this account, individual differences in cognitive or linguistic functions are expected to contribute to individual differences in the explicit processes required for recognition under adverse conditions.

Consistent with the ELU model, studies suggest that individual differences in cognition are associated with individual differences in the processing of speech under adverse listening conditions (e.g., Salthouse, 1994, 1996). For example, cognitive speed of processing contributes to the perception of both time-compressed speech (a form of rapid speech; Wingfield et al., 1985; Wingfield, 1996; Dias et al., 2019) and speech in noise (Tun and Wingfield, 1999). Working memory and attention (specifically, divided attention and selective listening) are also associated with perception of time-compressed speech (Tun et al., 1992; Vaughan et al., 2006) and speech in noise (Pichora-Fuller et al., 1995; Tun and Wingfield, 1999; Schneider et al., 2002, 2007; Tun et al., 2002). For dichotic speech, declines in attention are also related to performance declines (McDowd and Shaw, 2000; Rogers, 2000). Linguistic context can also positively contribute to speech perception (for review, see Burke and Shafto, 2008). Larger vocabulary in older adults and improved ability to utilize contextual cues facilitate speech perception in adverse listening conditions (Pichora-Fuller et al., 1995; Verhaeghen, 2003; Sheldon et al., 2008; Ben-David et al., 2015; Signoret and Ruder, 2019).

However, it is probably the combination of sensory and cognitive factors that affect speech perception of older adults (e.g., Cherry, 1953; Humes et al., 2006; Bronkhorst, 2015). If listeners possess a finite amount of information-processing resources (Kahneman, 1973), and if hearing-impaired older adults have to divert some of them to the normally automatic process of auditory encoding, then fewer resources will be available for subsequent higher-level processing (Rabbitt, 1990; Pichora-Fuller, 2003a). In addition, the interplay between sensory and cognitive factors can change in different listening conditions, but studies on the contribution of cognition to individual differences in speech perception in older adults often focused on a single task, making it hard to determine if either the contribution of cognition or the cognitive/sensory interplay changes across speech tasks. Whether the use of hearing aids changes, this interplay is also unknown.

### Rapid Perceptual Learning Accounts for Variance in Speech Perception in Older Adults

Rapid perceptual learning also relates to the variability in perception of speech under challenging conditions (Peelle and Wingfield, 2005; Golomb et al., 2007; Manheim et al., 2018; Banai and Lavie, 2020; Rotman et al., 2020b). Rapid perceptual learning, defined as the ability to rapidly adapt to changes in one’s environment, occurs under many adverse or sub-optimal conditions (Samuel and Kraljic, 2009). Perceptual learning is observed in old age, but it appears to be slower or reduced (Schneider and Pichora-Fuller, 2001; Forstmann et al., 2011; Lu et al., 2011) and more specific (Peelle and Wingfield, 2005) than in young adults (for a recent review, see Bieber and Gordon-Salant, 2021). Age-related hearing loss might have a further negative effect on learning. For example, older adults with preserved hearing exhibit poorer rapid learning of time-compressed speech compared to young adults, but better rapid learning than older adults with age-related hearing loss (Manheim et al., 2018). Relevant to the current study, across a range of speech tasks, rapid learning was documented in older adults with different levels of hearing (Peelle and Wingfield, 2005; Karawani et al., 2017; Manheim et al., 2018).

Perceptual learning and speech perception are related in the sense that learning contributes to future perception (Ahissar et al., 2009; Samuel and Kraljic, 2009; Banai and Lavie, 2021). However, recent studies suggest that the links could go beyond what could be expected from associations across different speech tasks (Banai and Lavie, 2021). Recent studies on perceptual learning (with both visual and speech materials) suggest that a general learning factor across learning tasks could serve as an individual capacity that supports performance across a range of scenarios (Yang et al., 2020; Dale et al., 2021; Heffner and Myers, 2021). Consistent with this view, we observed that individual differences in rapid perceptual learning of one type of speech (e.g., time-compressed speech) are consistently related to individual differences in speech perception under different adverse conditions (speech in noise and fast speech; Karawani et al., 2017;Manheim et al., 2018; Rotman et al., 2020b). Speech perception and rapid learning have also been found to be associated even when learning is assessed under conditions designed to offset the effects of age and hearing loss on speech perception (Manheim et al., 2018; Rotman et al., 2020b). We hypothesize that individuals who retain good rapid perceptual learning despite aging and hearing loss, can offset some of their negative impacts through rapid online learning (Banai and Lavie, 2021). To further explore this hypothesis, we now focus on the unique contribution of rapid perceptual learning to other challenging listening conditions, after accounting for sensory and cognitive factors and for the use of hearing aids.

### Hearing Aid Use

For older adults with hearing loss, hearing aids are the most widely used rehabilitation devices. While hearing aids are unlikely to fully compensate for the auditory processing deficits of individuals with hearing impairment, they amplify sounds to improve audibility (Souza, 2016) and incorporate multiple algorithms intended to improve communication in adverse listening conditions (Neher et al., 2014). However, the perceptual results of using hearing aids depend not only on hearing aid technology but also on the factors described above. Moreover, long-term acclimatization induced benefits may further improve speech perception in some individuals. The effects reported in the literature include improved speech perception in noise, reduced distractibility to background noise, and reduced listening effort (Gatehouse, 1992; Munro and Lutman, 2003; Lavie et al., 2015; Habicht et al., 2016; Dawes and Munro, 2017; Lavie et al., 2021). However, other studies have failed to demonstrate improved identification of degraded speech (speech in noise, Dawes et al., 2013, 2014a,b; fast speech, Rotman et al., 2020a) in new or experienced hearing aid users. Thus, even though there are some indications for perceptual gains after months or years of hearing aid use, the effects of hearing aids on higher-level language processes in complex listening conditions are not well understood.

Unsurprisingly, most studies seeking to explain individual differences in aided speech perception have identified differences in hearing thresholds as the main source of variance (e.g., Tun and Wingfield, 1999; Humes, 2007). However, after controlling for the effects of audibility and age, working memory span score was correlated with both aided and unaided perception of speech in noise (Lunner, 2003), and the benefit from hearing aid algorithms (i.e., fast acting compression) was positively associated with cognitive skill (Lunner, 2003; Gatehouse et al., 2006; Cox and Xu, 2010; Souza et al., 2015).

### Research Questions and Hypotheses

According to the literature review above, the interplay between the perception of different types of degraded speech, multiple cognitive factors, and perceptual learning is not sufficiently understood. It is also unclear whether the use of hearing aids changes the interplay among the different factors or results in plastic changes in speech perception (see Lavie et al., 2021 for a systematic review). The present study was designed to address these issues by investigating the contribution of hearing, cognition, rapid perceptual learning, and the contribution of long-term hearing aid use to three indices of speech perception: fast speech, speech in babble noise, and dichotic speech.

If, as explained above, perceptual learning is a capacity that can support other processes, such as “online” speech perception, rapid perceptual learning should explain unique variance in the perception of different types of distorted speech in addition to the known contributions of other sensory and cognitive factors. To this end, we use rapid learning of time-compressed speech as an index of learning for two reasons. First, the work reviewed above suggests that with this task, rapid learning rates are maintained even in older adults with hearing loss. Second, most listeners have no experience with this form of accelerated speech, and initial performance can be quite poor, making it easy to observe learning. Additionally, we hypothesize that the same factors that account for individual differences in degraded speech processing in adults with presbycusis also play a role when it comes to the effects of hearing aids, but the current literature (see Kalluri et al., 2019; Lavie et al., 2021 for recent reviews) makes it hard to draw more specific hypotheses, and therefore, in this regard, this is an exploratory study.

## Materials and Methods

### Participants

A total of 95 potential participants were recruited *via* hearing clinics, retirement communities, and community centers. Potential participants were screened based on the following inclusion criteria: (1) age 65 and above; (2) bilateral, adult-onset, symmetric, sensory hearing loss of 30–70 dB, with flat or moderately sloping audiograms, and suprathreshold word recognition scores of ≥60% and air-bone gaps ≤15 dB; (3) no known neurological or psychiatric diagnoses; (4) normal or corrected-to-normal vision; (5) high proficiency in Hebrew; (6) normal cognitive status [a score of 24 or higher on the Mini-Mental State Examination (MMSE; Folstein et al., 1975)]; and (7) hearing aid use: we targeted only non-users (no prior experience with hearing aids and no plans to acquire hearing aids during the period of the study) and experienced hearing aid users [at least 6 months of bilateral hearing aid use; hearing aids were digital, with at least 16 amplification channels, at least four compression channels, noise reduction and anti-feedback algorithms, and wireless (ear to ear) processing]. Participants received modest monetary compensation for their participation and signed written informed consent forms. All aspects of the study were approved by the ethics committee of the Faculty of Social Welfare and Health Sciences at the University of Haifa (permit 362/18).

Twenty-two recruits failed to meet inclusion criteria and were excluded from the study: 12 for having insufficient hearing loss, five for having more severe hearing loss or low suprathreshold word recognition scores, two for asymmetric hearing loss, two for having insufficient experience with hearing aids [in the experienced hearing aid group (see below)], and one for reporting additional motor issues that could have influenced their responses on some of the tasks (e.g., block design and flanker). Three additional participants completed the first experimental session only (see experimental design below), and their data were thus excluded from all analyses.

**Figure 1:**
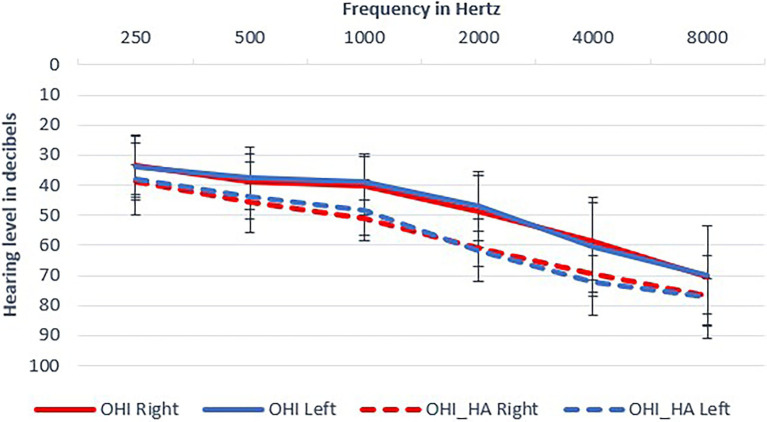
Mean audiograms of participants. Mean thresholds and standard deviations are shown: older hearing-impaired adults (OHI) in full lines; older hearing-impaired adults who use hearing aids (OHI-HA) in dashed lines.

The final study sample included 70 participants (23 males and 47 females) who met all inclusion criteria: 35 older adults with hearing loss (OHI) who did not use hearing aids and 35 older hearing-impaired adults who were experienced hearing aid users (OHI-HA). The two groups had similar ages, MMSE and cognitive scores, but hearing aid users had poorer hearing, somewhat poorer suprathreshold word recognition scores and somewhat higher education (see Figure 1; Table 1). Hearing thresholds were considered in our statistical modeling; the differences in word recognition (corresponding to 1–2 words difference) and education were considered negligible. Based on a power analysis on the data of our previous study (Rotman et al., 2020b), no effect for hearing aid use was expected even if we increased our sample size to 400 (200 in each group) participants, which was unrealistic. In contrast, a sample of 40 participants (20 in each group) was deemed sufficient to replicate the perceptual, learning, and cognitive effects reported by Rotman et al. (2020a) with a statistical power of 0.8. Power calculation was performed using the *simr* package (Green et al., 2016) in R.

**Table 1:** Age, hearing, word recognition, education, and cognitive screening.

	OHI	OHI-HA
**Age (years)>**		
Mean (SD) [95% CI]	79 (7) [77–82]	81 (6) [78–83]
Median (IQR)	81 (73–84)	80 (76–85)
**Hearing (PTA4, dB)>**		
Mean (SD) [95% CI]	46 (7) [43–49]	57 (8) [53–59]
Median (IQR)	44 (40–52)	56 (53–61)
**Suprathreshold word recognition scores>**		
Mean (SD) [95% CI]	90 (9) [86–93]	84 (9) [81–87]
Median (IQR)	92 (86–95)	85 (77–90)
**Years of education>**		
Mean (SD) [95% CI]	14 (3) [13–15]	16 (4) [14–17]
Median (IQR)	14 (12–15.5)	16 (12.5–18)
**MMSE>**		
Mean (SD) [95% CI]	28 (1) [27–28]	28 (1) [28–29]
Median (IQR)	28 (27–29)	28 (28–29)

*PTA, Pure-tone average; MMSE, Mini-mental state examination; CI, Confidence interval; IQR, Interquartile range; OHI, Hearing-impaired older adults; and OHI-HA, Hearing-impaired older adults who are experienced hearing aid users*.

### Procedure

**Figure 2:**
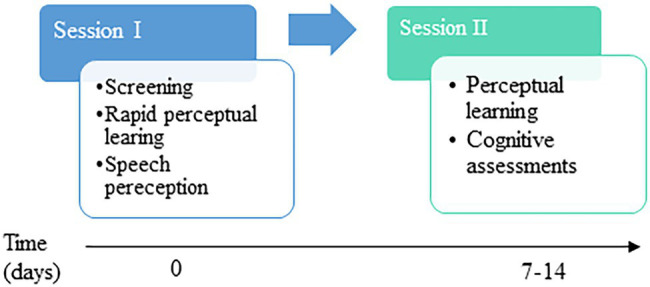
Schematic illustration of study design.

Testing was comprised of two sessions conducted 7–14 days apart at a hearing clinic or at the participants’ home, based on each participant’s preference (see Figure 2). Except for the audiometric assessments in the clinic (see below), all other testing was conducted in a quiet room in the clinic or in the participants’ homes. In session I, potential participants were screened based on the inclusion criteria. Participants who met the inclusion criteria underwent assessments of rapid perceptual learning of time-compressed speech, perception of fast speech, and speech in noise and dichotic word identification. Session II included cognitive assessments and another assessment of time-compressed speech learning, data from which are not reported here. All testing was conducted by two clinical audiologists experienced in working with hearing-impaired patients and therefore accustomed to speaking loudly and clearly.

### Task

#### Screening Assessments

##### Demographic Questionnaire

A questionnaire regarding education, handedness, lifestyle, and general health was used in the current study. The participants completed the questionnaire before completing further assessments.

##### Cognitive and Hebrew Screening

Participants were screened with a the MMSE (Folstein et al., 1975), with a cutoff score of 24 as an inclusion criterion. Proficiency in Hebrew was evaluated using a short screening with a series of questions and commands in Hebrew. To participate in the study, one had to complete this screening with a perfect score (see Lavie, 2011).

##### Audiological Assessments

A full pure-tone and speech audiometry (suprathreshold word recognition) was conducted in an acoustic booth, using the MAICO audiometer (model MA42) or at the participants’ home with Inventis *Cello* and Piccolo portable audiometers and Silenta Supermax supra-aural headphones. Most comfortable levels (MCL) for speech were also assessed. The audiograms were classified based on Duthey (2013), with four frequencies pure-tone average (0.5, 1, 2, and 4 KHz) ≥ 30 dB as a criterion of hearing loss. Participants with up-to-date (≤6 months) audiograms were not evaluated again.

#### Speech Perception and Learning

##### Stimuli

Stimuli were 80 simple sentences in Hebrew, five to six words long, with a common subject-verb-object grammatical structure (adapted from Prior and Bentin, 2006). All sentences were recorded in a sound attenuating booth using a built-in MacBook Air microphone, sampled at 44 KHz and saved in WAV format. The root-mean-square levels of the recorded sentences were normalized using Audacity audio software version 2.2.0. Sentences were recorded by four native Hebrew speakers (three females and one male). Speaker 1 (female) recorded 10 different sentences at her natural fast rate (*M* = 183 words/min, *SD* = 17); speaker 2 (female) recorded 10 sentences at her natural fast rate (*M* = 210 words/min, *SD* = 21) and 10 sentences at her normal, unhurried rate (*M* = 111 words/min, *SD* = 27); speaker 3 (male) recorded 10 sentences at a normal rate of 88 words/min (*SD* = 10.30); and speaker 4 (female) recorded 40 sentences at a normal rate of 102 words/min (*SD* = 12.68). To minimize the effects of sentence familiarity on performance, there was no sentence repetition within or across conditions. In addition, Speaker 4 also recorded a list containing 25 pairs of monosyllabic words, adapted from the Hebrew PB-50 test (Lavie et al., 2015) for the dichotic word identification task (see below).

##### Presentation and Scoring

Speech materials were presented through Meze 99 classics headphones to both ears as follows: (1) unaided to the OHI group and (2) aided for the OHI-HA group (i.e., headphones were placed while participants wore their hearing aids). Stimuli were presented at each listener’s preferred level. To determine this level, a pre-recorded short passage was played and listeners determined their preferred listening level. Because some of the participants were tested at home, and others in several rooms in the clinic, achieving constant acoustic settings for sound field presentation of the speech stimuli was impossible. Thus, we decided to test all participants with headphones and play the stimuli from the computers (in line with Rotman et al., 2020b). In the OHI-HA group, the testers verified that the hearing aids were working properly at the beginning of each session. After listening to each test stimulus (sentences or dichotic word pairs), participants were asked to repeat what they had heard, and the experimenter transcribed their replies. Each stimulus was presented only once, and no feedback was provided. Performance was scored off-line. For the rapid learning, fast speech and speech in noise tasks, all words, including function words, were counted for scoring. Scoring of the dichotic listening task is described below. Unless otherwise noted, the proportion of correctly recognized words/sentence was computed and used for statistical modeling, although for visualization proportion was averaged across sentences.

##### Rapid Perceptual Learning (Session I)

Ten sentences (recorded by Speaker 2) were presented as time-compressed speech. Time-compressed speech was chosen because learning with this form of speech was previously documented in older adults within and across sessions (e.g., Peelle and Wingfield, 2005; Golomb et al., 2007; Manheim et al., 2018; Rotman et al., 2020b). In addition, most older listeners have no experience with this type of artificially accelerated speech, making it useful in studying the correlations between learning and the recognition of other forms of degraded speech (e.g., naturally fast speech and speech in noise). Following earlier work (Rotman et al., 2020b), sentences were compressed to 45–50% of their original length (45% for participants with PTA of 26–47 dB and 50% for PTA ≥ 48 dB) in Matlab, using a pitch preserving algorithm (WSOLA, Verhelst and Roelands, 1993). Speech rates were adjusted based on hearing threshold to minimize the effects of hearing on the estimate of rapid learning.

Baseline recognition of time-compressed speech was defined as the proportion of correctly identified words in the two first sentences. Learning of time-compressed speech was defined as the rate of improvement in recognition over time. It was quantified as the linear slopes of the learning curves over an additional eight time-compressed speech sentences (for further details see Rotman et al., 2020b).

##### Speech Perception

Speech perception was evaluated using the following tasks:

###### Fast Speech

Twenty sentences (10 sentences recorded by Speaker 1 and 10 sentences recorded by Speaker 2).

###### Speech Recognition in Noise

Twenty sentences were presented (10 sentences recorded by Speaker 3 and 10 sentences recorded by Speaker 4). All sentences were embedded in a 4-talker babble noise with a fixed SNR level of +3 dB.

###### Dichotic Word Identification

Following previous research (Lavie et al., 2013, 2015), we used a list of 25 pairs of monosyllabic words, adapted from the Hebrew PB-50 test. One word of each pair was presented to the right ear while the other word was presented simultaneously to the left ear, and participants were required to repeat both words in whichever order they chose. For statistical analysis, the number of correctly repeated words in each ear was counted and two indices of dichotic listening were calculated as: the sum (= dominant ear score + non-dominant ear score) and the difference between the ears (= dominant ear - non-dominant ear).

#### Cognitive Assessments

A battery of cognitive assessments was used to evaluate cognitive status and identify characteristics that might influence participants’ performance on the experimental tasks. This battery was administered at a comfortable auditory level that was defined by each participant to negate potential confounding effects of audibility on performance. The following subtests from the Wechsler Adult Intelligence Scale-Third Edition in Hebrew (WAIS-III; Wechsler, 1997) were used as: **vocabulary** (semantic knowledge), **digit span** (working memory), and **block design** (non-verbal reasoning).

All subtests were administrated and scored according to the test manual. Raw scores were converted to standardized scores.

##### Attention

Two tests were used as: (1) **Flanker test** (Eriksen and Eriksen, 1974). A computerized version of the well-validated Flanker test was used as a measure of inhibition and selective attention. The target stimulus was an arrow-head heading right or left, embedded in the middle of a row of five arrow-heads or other stimuli. Participants were asked to note the direction of a central arrow, which was flanked by arrows pointing in the same direction (congruent trials) or the opposite direction (incongruent trials) or non-arrow stimuli (neutral trials). Reaction time and accuracy were measured. The “flanker cost” for each participant was used for statistical analyses. The cost was calculated as the mean logRT.

(RT = reaction time in ms) of the correct responses in the incongruent trials divided by the mean log RT of the correct responses in the neutral trials. A higher flanker cost (>1) means poorer selective attention. (2) **Trail making test** (Reitan, 1958). Attention switching control was tested in two test conditions: in condition A, participants were asked to draw lines to connect circled numbers in a numerical sequence (i.e., 1-2-3) as rapidly as possible. In condition B, participants were asked to draw lines to connect circled numbers and letters in an alternating numeric and alphabetic sequence (i.e., 1-A-2-B) as rapidly as possible. Response speed was measured by a stopwatch.

## Results

### Descriptive Statistics

As shown in Table 2, hearing aid users had somewhat higher vocabulary scores than the non-hearing aid group, and this was considered in the statistical analyses reported below. In both groups, there was large between-participant variance across all speech and learning tasks (the raw data and analysis code can be found at https://osf.io/sreq4).

**Table 2:** Cognition and speech perception.

	OHI	OHI-HA
**Cognition**		
Vocabulary (scaled score)		
Mean (SD) [95% CI]	11.6 (2.4) [11–12]	13.9 (2.6) [13–15]
Median (IQR)	12 (10–13)	14 (12–16)
Working memory (scaled score)		
Mean (SD) [95% CI]	9.3 (2.2) [8–10]	10.5 (3.1) [9–11]
Median (IQR)	9 (7–10)	10 (8–12)
Block design (scaled score)		
Mean (SD) [95% CI]	10.9 (3.3) [10–12]	11.6 (4.1) [10–13]
Median (IQR)	11 (9–13)	10 (8–15)
Trail Making		
Mean (SD) [95% CI]	2.7 (1.0) [2.4–3.1]	2.4 (0.8) [2.1–2.7]
Median (IQR)	2.4 (2.0–3.7)	2.2 (1.8–2.8)
Flanker cost		
Mean (SD) [95% CI]	1.01 (0.01) [1.01–1.02]	1.02 (0.02) [1.01–1.02]
Median (IQR)	1.01 (1.01–1.02)	1.01 (1.01–1.02)
**Speech perception>**		
FS (proportion correct)		
Mean (SD) [95% CI]	0.28 (0.19) [0.22–0.35]	0.22 (0.16) [0.16–0.27]
Median (IQR)	0.32 (0.04–0.62)	0.20 (0.02–0.60)
SIN (proportion correct)		
Mean (SD) [95% CI]	0.68 (0.20) [0.62–0.75]	0.50 (0.22) [0.42–0.58]
Median (IQR)	0.74 (0.03–0.96)	0.52 (0.07–0.96)
Dichotic listening (sum)		
Mean (SD) [95% CI]	0.63 (0.30) [0.53–0.73]	0.55 (0.22) [0.48–0.63]
Median (IQR)	0. 6 (0.16–1.32)	0.52 (0.24–1.08)
Dichotic listening (gap)		
Mean (SD) [95% CI]	0.21 (0.12) [0.17–0.26]	0.18 (0.12) [0.14–0.23]
Median (IQR)	0.24 (0–0.56)	0. 2 (0–0.4)
TCS baseline (proportion correct)		
Mean (SD) [95% CI]	0.158 (0.19) [0.11–0.20]	0.151 (0.13) [0.11–0.19]
Median (IQR)	0.09 (0–0.73)	0.18 (0–0.73)
TCS learning slope		
Mean (SD) [95% CI]	0.095 (0.07) [0.07–0.12]	0.094 (0.07) [0.07–0.12]
Median (IQR)	0.086 (−0.01–0.30)	0.090 (−0.002–0.22)

*FS, Fast speech; SIN, Speech in noise; TCS, Time-compressed speech; PTA, Pure-tone average; CI, Confidence interval; IQR, Interquartile range; OHI, Older hearing-impaired adults; and OHI-HA, Older hearing-impaired adults who are experienced hearing aid users*.

As shown in Table 3, rapid perceptual learning of time-compressed speech was positively correlated with identification of fast speech and speech in noise, and negatively correlated with hearing thresholds. In addition, and as expected from the literature, speech perception was correlated with specific cognitive indices. Rapid learning of time-compressed speech also correlated with some of the cognitive measures.

**Table 3:** Correlations between speech perception, cognition, and learning among all participants.

	FS	SIN	Dichotic listening (sum)	Dichotic listening (gap)	Slope
Hearing	−0.50	−0.59	−0.35	−0.16	−0.38
Vocabulary	0.10	−0.03	−0.008	−0.11	0.11
Working memory	**0.36>**	**0.33>**	0.24	−0.004	**0.28>**
Flanker cost	−0.05	−0.13	0.07	0.15	−0.18
TCS baseline	**0.56>**	**0.49>**	0.11	−0.05	**0.35>**
Slope	**0.54>**	**0.46>**	0.23	0.06	–

*Pearson correlations are shown. FS, Fast speech; SIN, Speech in noise; hearing = average PTA; and TCS baseline = average of first two sentences of time-compressed speech. Vocabulary and working memory = raw scores from corresponding tests; Slope = rapid perceptual learning slope, session I. Bold entries represent significant correlations (p < 0.05) after correcting for multiple testing with a Bonferroni correction*.

### Modeling Speech Perception As a Function of Age, Hearing, Cognition, Rapid Perceptual Learning, and Hearing Aid Use

The contribution of hearing and cognition to recognition accuracy in the speech tasks was studied in the past. Therefore, our modeling here focused on the unique additional contributions of perceptual learning and hearing aid use. To this end, modeling was performed in stages: hearing and cognition were modeled first, followed by learning, and then hearing aid use. With this approach, if a later model fits the data significantly better than a previous one (with a model comparison), the predictor entered at the later stage has a unique contribution to speech recognition when all other included variables are considered. Within a given model, the coefficient of each predictor reflects its contribution while all other predictors in the model are kept constant. Since there were repeated measures for the fast speech and speech in noise, a series of generalized linear mixed models was run using the lme4 package in R (Bates et al., 2014). Single trial fast speech and speech in noise scores served as the dependent variables, and age, hearing, cognition, rapid perceptual learning, and hearing aid use served as the independent variables (i.e., the predictors). Given the number of predictors relative to sample size, and to avoid overloading the models, block design and trail making were excluded from the analysis; likewise, interactions were not modeled. The random effects structure consisted of random intercepts for both participant and sentence; predictors were standardized (z-scored) prior to modeling. Following earlier work, and due to dealing with proportion scores, binomial regressions with a logit link function (logistic regressions) were used (Rotman et al., 2020b).

Five models were constructed for fast speech and for speech in noise, starting with a model that included only the random effects (Model 0). Thereafter, each subsequent model added one additional predictor over the previous model(s), with the models building upon one another sequentially (e.g., model 1 = Model 0 + variable 1; Model 2 = Model 1 + variable 2; and Model 3 = Model 2 + variable 3). Model 1 included background variables of the participants as predictors, which included as: age, hearing, vocabulary, working memory, and attention. Model 2 included baseline recognition of time-compressed speech; Model 3 added the rapid perceptual learning slope; and Model 4 added hearing aid use (rated on a nominal scale—yes/no). To isolate the unique contribution of each additional variable, these four increasingly complex models were compared using likelihood ratio tests with the R ANOVA function.

Note that in general, correlations between the different predictors were not high (the highest Pearson correlations were *r* = 0.43 between vocabulary and working memory, *r* = −0.38 between hearing and learning, and *r* = 0.35 between learning and baseline recognition of TCS), suggesting that multicollinearity is not a serious concern. Likewise, all Variance Inflation Factors (VIF) were low (< 2), as reported below for the best fitting models.

#### Recognition of Fast Speech

The inclusion of the background variables in the model resulted in a better fit to the data than the model that included the random effects only. However, the addition of baseline recognition of time-compressed speech and rapid learning both improved the fits significantly, suggesting that rapid learning had a significantly unique contribution to the recognition of fast speech, beyond that of other variables. Hearing aids had no additional effect (see Table 4).

**Table 4:** Fast speech—model comparisons.

Model	Fixed effects	AIC	***χ*** ^2^	*Df*	*p*
0	(Random effects)	3630.7	–	–	–
1	+ Background variables	3595.5	45.18	5	< 0.001
2	+ Baseline recognition of TCS	3581.7	15.76	1	< 0.001
3	+ Rapid learning slope	3576.4	7.32	1	0.007
4	+ Hearing aids	3578.4	0.03	1	0.868

*As described in the main text, the initial model included age, hearing, vocabulary, working memory, and attention as predictors. Comparison models successively added the fixed effects of baseline recognition of time-compressed speech, rapid perceptual learning slope, and hearing aids. The random effects structure was identical across models*.

**Figure 3:**
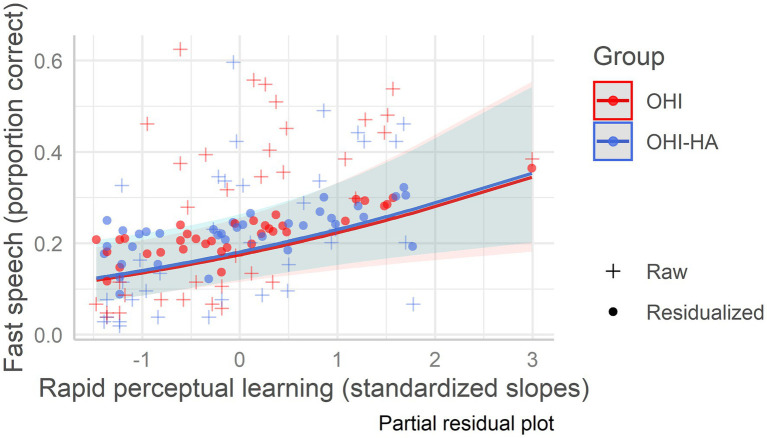
Fast speech recognition as a function of rapid learning among older hearing-impaired adults and older hearing-impaired adults who use hearing aids. Older hearing-impaired adults (OHI) in red; older hearing-impaired adults who use hearing aids (OHI-HA) in blue. The *y*-axis indicates the correct perception percentage of fast speech, and the *x*-axis indicates the standardized rapid perceptual learning slope. The dots (residualized aggregate scores) mark the predicted scores; their deviation from the regression line indicates prediction error, while the pluses mark the raw/true scores. The shaded areas are the confidence intervals.

In the best fitting model (model 3), age, hearing, baseline recognition of time-compressed speech, and learning were all significant predictors of fast speech recognition (see Table 5 which also includes model 1 with only background variables). Hearing was the strongest negative predictor (largest beta in absolute value, see Table 5) of fast speech recognition followed by age, indicating that fast speech recognition was poorer in individuals with more severe hearing loss and in older individuals. Baseline recognition of time-compressed speech and rapid learning were both positive predictors, suggesting that for a given age/hearing loss, listeners who maintained better perception and learning of time-compressed speech also maintained more accurate recognition of fast speech, regardless of hearing aid use (see Figure 3). Variance Inflation Factors for the best fitting model were 1.27 for age, 1.91 for hearing, 1.41 for vocabulary, 1.55 for working memory, 1.06 for attention, 1.31 for baseline recognition of TCS, and 1.46 for learning.

**Table 5:** Results of generalized linear mixed-model for fast speech recognition as a function of the background variables (Model 1) and as a function of age, hearing, cognition, baseline recognition of time-compressed speech, and rapid perceptual learning as fixed effects (Model 3).

Fixed effect	Odds ratio	*β*	*SE*	95% CI	*Z*	*p*
Model 1						
Age	0.73	−0.32	0.12	[0.57, 0.92]	−2.63	0.009
Hearing (PTA4)	0.53	−0.64	0.12	[0.41, 0.67]	−5.28	<0.001
Vocabulary	1.21	0.22	0.13	[0.97, 1.59]	1.69	0.091
Working memory	1.32	0.28	0.13	[1.02, 1.70]	2.14	0.033
Attention	1.00	−0.002	0.12	[0.79, 1.26]	−0.02	0.984
Model 3						
Age	0.79	−0.24	0.10	[−0.44, −0.03]	−2.26	0.023
Hearing (PTA4)	0.62	−0.48	0.11	[−0.70, −0.27]	−4.35	<0.001
Vocabulary	1.15	0.14	0.11	[−0.07, 0.36]	1.30	0.219
Working memory	1.10	0.10	0.12	[−0.13, 0.32]	0.83	0.414
Attention	1.03	0.03	0.10	[−0.18, 0.23]	0.25	0.803
Baseline TCS	1.47	0.39	0.10	[0.18, 0.59]	3.68	<0.001
Learning	1.36	0.31	0.11	[0.09, 0.52]	2.77	0.008

*PTA, Pure-tone average; TCS, Time-compressed speech; and CI, Confidence interval*.

#### Recognition of Speech in Noise

The inclusion of the background variables in the model resulted in a better fit to the data than the model that included the random effects only (Table 6). However, baseline recognition of time-compressed speech improved the fits significantly (see Table 6), suggesting that time-compressed speech perception had a significant unique contribution to the recognition of speech in noise, beyond that of other variables. Hearing aids had no additional effect.

**Table 6:** Speech in noise—model comparisons.

Model	Fixed effects	AIC	*χ* ^2^	*df*	*p*
0	(Random effects)	4454.3	–	–	–
1	+ Background variables	4417.5	46.79	5	0.000
2	+ Baseline recognition of TCS	4406.7	12.75	1	0.000
3	+ Rapid learning slope	4408.1	0.62	1	0.43
4	+ Hearing aids	4407.4	2.73	1	0.09

*Model 1 included age, hearing, vocabulary, working memory, and attention as predictors. Comparison models successively added the fixed effects of baseline recognition of time-compressed speech, rapid perceptual learning slope, and hearing aids. The random effects structure was identical across models*.

**Figure 4:**
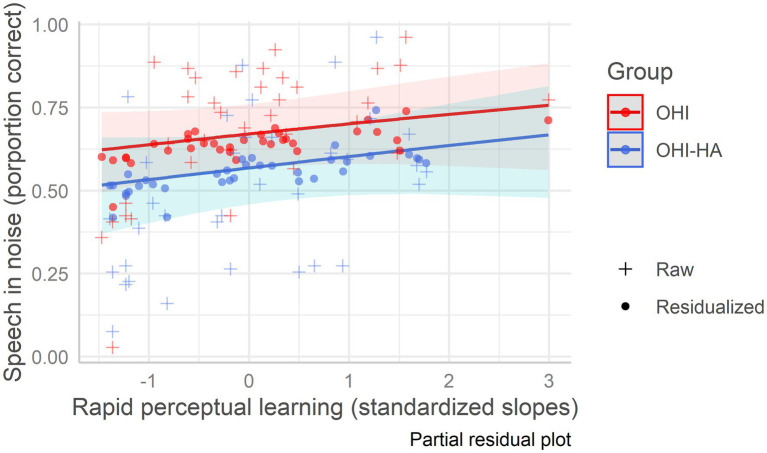
Speech in noise recognition as a function of rapid learning among older hearing-impaired adults and older hearing-impaired adults who use hearing aids. Older hearing-impaired adults (OHI) in red; older hearing-impaired adults who use hearing aids (OHI-HA) in blue. The *y*-axis indicates the correct perception percentage of speech in noise, and the *x*-axis indicates the standardized rapid perceptual learning slope. The dots (residualized aggregate scores) mark the predicted scores; their deviation from the regression line indicates prediction error, while the pluses mark the raw/true scores. The shaded areas are the confidence intervals.

In the best fitting model (see Table 7 which also includes model 1 with only background variables), hearing was the strongest predictor (i.e., largest beta in absolute value) of speech in noise recognition, followed by baseline recognition of time-compressed speech. Neither rapid learning nor hearing aid use further improved the fit (see Figure 4). Thus, lower hearing thresholds and more accurate time-compressed speech recognition were associated with better recognition of speech in noise. Variance Inflation Factors for the best fitting model were 1.29 for age, 1.97 for hearing, 1.45 for vocabulary, 1.55 for working memory, 1.06 for attention, 1.33 for baseline recognition of TCS, and 1.53 for learning.

**Table 7:** Results of generalizedlinear mixed-effects model for speech in noise recognition as a function of the background variables (Model 1) and as a function of age, hearing, cognition, and baseline recognition of time-compressed speech as fixed effects (Model 2).

Fixed effect	Odds ratio	*B*	*SE*	95% CI	*Z*	*p*
Model 1						
Age	0.79	−0.24	0.12	[−0.48, 0.01]	−1.90	0.057
Hearing (PTA4)	0.49	−0.71	0.12	[−0.95, −0.47]	−5.86	<0.001
Vocabulary	1.07	0.07	0.13	[−0.19, 0.32]	0.51	0.607
Working memory	1.41	0.35	0.13	[0.09, 0.61]	2.61	0.009
Attention	0.95	−0.05	0.11	[−0.27, 0.17]	−0.43	0.668
Model 2						
Age	0.85	−0.17	0.11	[−0.39, 0.06]	−1.45	0.147
Hearing (PTA4)	0.51	−0.67	0.11	[−0.89, −0.45]	−6.04	<0.001
Vocabulary	1.04	0.04	0.12	[−0.19, 0.27]	0.34	0.731
Working memory	1.23	0.21	0.13	[−0.04, 0.45]	1.65	0.099
Attention	0.94	−0.06	0.10	[−0.26, 0.14]	−0.62	0.533
Baseline TCS	1.53	0.42	0.11	[0.20, 0.65]	3.74	<0.001

*PTA*, *Pure-tone average; TCS, Time-compressed speech; and CI*, *Confidence interval*.

#### Dichotic Word Identification

Since there were no repeated measures (i.e., there was only one score for each participant), linear regression analyses were used. Four models were constructed for the dichotic listening task in two different ways: once with the dichotic sum serving as the dependent variable and once with the dichotic gap serving as the dependent variable. The models all included age, hearing, vocabulary, working memory, and attention as predictors. Thereafter, as with the models mentioned above, each subsequent model added one additional variable, with the models building upon one another sequentially. Model 2 included baseline recognition of time-compressed speech; Model 3 added the rapid learning slope; and Model 4 added hearing aids. To isolate the unique contribution of each additional variable, these four successively complex models were compared using an ANOVA Table for Comparison of Nested Model tests.

Dichotic Sum as the Dependent Variable. For the dichotic listening task, with dichotic sum serving as the dependent variable, model comparisons showed that there were no contributions of variables/effects that did not appear in the first model (see Table 8).

**Table 8:** Dichotic sum—results of the comparison of nested model tests.

Model	Fixed effects	AIC	Res. *df*	RSS	*df*	Sum of Sq.	*F*	*p*
1	Background variables	195.724	64	54.964				
2	+ Baseline recognition of TCS	197.715	63	54.957	1	0.001	0.01	0.93
3	+ Rapid perceptual learning slope	199.299	62	54.631	1	0.326	0.36	0.55
4	+ Hearing aids	201.293	61	54.627	1	0.004	0.00	0.94

*As described in the main text, the initial model included age, hearing, vocabulary, working memory, and attention as predictors. Comparison models successively added the fixed effects of baseline recognition of time-compressed speech, rapid perceptual learning slope, and hearing aids. The random effects structure was identical across models*.

##### Dichotic Gap As the Dependent Variable

For the dichotic listening task, with dichotic gap serving as the dependent variable, model comparisons showed that there were no contributions of variables/effects that did not appear in the first model (see Table 9).

**Table 9:** Dichotic gap—results of the comparison of nested model tests.

Model	Fixed effects	AIC	Res. *df*	RSS	*df*	Sum of Sq.	*F*	*p*
1	Background variables	207.182	64	64.739				
2	+ Baseline recognition of TCS	208.607	63	64.210	1	0.530	0.51	0.48
3	+ Rapid perceptual learning slope	210.328	62	63.953	1	0.256	0.24	0.62
4	+ Hearing aids	212.189	61	63.827	1	0.127	0.12	0.73

*As described in the main text, the initial model included age, hearing, vocabulary, working memory, and attention as predictors. Comparison models successively added the fixed effects of baseline recognition of time-compressed speech, rapid perceptual learning slope, and hearing aids. The random effects structure was identical across models*.

## Discussion

We assessed the relative contribution of hearing acuity, cognitive factors, and rapid perceptual learning to the identification of fast speech, speech in noise, and dichotic speech in older adults with hearing loss. Hearing acuity and time-compressed speech perception uniquely contributed to the perception of both fast speech and speech in noise. Rapid perceptual learning was a significant predictor of fast speech perception even after accounting for age, hearing, and cognition. Hearing aid use had no effect on any of the speech tasks. Our findings suggest that in older adults, good rapid perceptual learning can partially offset the effects of age and hearing loss on the perception of fast speech, but not on the perception of speech in noise or dichotic speech. Determining if this is due to inherent differences between the different speech tasks or due to other differences (e.g., overall level of difficulty) requires further investigation. Furthermore, the finding that time-compressed speech recognition is strongly associated with the perception of speech in noise suggests a potential link between the perception of these two types of challenging speech.

In the present study, hearing acuity was the strongest predictor of both fast speech and speech in noise perception. This finding is consistent with previous work on speech perception in older adults (e.g., Frisina and Frisina, 1997; Janse, 2009; Humes and Dubno, 2010). For example, Janse (2009) investigated the relative contributions of auditory and cognitive factors to fast speech perception in older adults. While hearing acuity, reading rate, and visual speed of processing were all significant predictors, hearing acuity was the strongest one. Similarly, for speech in noise among new and experienced hearing aid users, hearing loss was repeatedly identified as the primary and best predictor for unaided performance (Humes, 2002). Our study extends this finding to the perception of fast speech among hearing aid users.

An interesting outcome of the current study is that the initial performance of time-compressed speech remained the second strongest predictor of perception of both fast speech and speech in noise. These findings are in line with previous results regarding the perception of fast speech (Manheim et al., 2018; Rotman et al., 2020b) and extend them to speech in noise. Although fast speech is harder to recognize than time-compressed speech at similar rates, performance is correlated between these two tasks, and temporal processing is likely involved in the perception of both (Janse, 2004; Gordon-Salant et al., 2014). Indeed, the increased difficulties older adults have in processing distorted speech are thought to result in part from age-related declines in temporal processing (e.g., Pichora-Fuller and Singh, 2006; Anderson et al., 2011; Füllgrabe et al., 2015). Temporal cues within both the temporal envelope of the speech signal and its fine structure convey information that influences lexical, syntactic, and phonemic processing and these can support speech perception across a range of conditions (Kidd et al., 1984; Nelson and Freyman, 1987; Festen and Plomp, 1990; Rosen, 1992). Fast speech recognition can thus be affected by the temporal resolution of phonetic information and by linguistic context, suggesting that both low-level and high-level processes can independently contribute to the processing of temporally distorted speech (Gordon-Salant and Fitzgibbons, 2001; Pichora-Fuller, 2003b; Gordon-Salant et al., 2014).

As for the association between time-compressed speech and speech in noise recognition, loss of synchrony in aging auditory systems may disrupt the fine structure cues that important for recognizing speech in noise (Schneider and Pichora-Fuller, 2001). The fine structure of speech, in particular its harmonic structure, enables listeners to attend to a target speech source or to distinguish competing speech or noise sources, especially when they are spectrally similar to the target signal (Moore, 2008, 2011). Similarly, binaural advantage for detecting and identifying speech presented in a noisy background relies on the ability of the binaural system to process interaural, minimal timing differences (Levitt and Rabiner, 1967). If the perception of temporal fine structure affects both identification of speech in the presence of competing noise and fast speech, it is perhaps unsurprising that perception of time-compressed speech accounts for some of the individual differences in the perception of speech in noise. Indeed, speech reception threshold in fluctuating noise and susceptibility to time compression are highly correlated among normal-hearing and hearing-impaired older adults (Versfeld and Dreschler, 2002).

Our results indicate that the association across speech tasks is not limited to tasks that share obvious sensory characteristics. This suggests that common speech perception processes could underlie performance variability across a range of listening challenges in older adults with different levels of hearing. Consistent with this view, research on speech recognition under adverse listening conditions has shown relationships across different conditions (e.g., Borrie et al., 2017; Carbonell, 2017). For example, Carbonell (2017) found that performance was correlated across noise-vocoded, time-compressed, and speech in babble noise tasks, and regression models that predicted performance on one task based on performance of the other two also showed a strong relationship. Nevertheless, it is hard to determine whether these findings reflect common underlying processing. Furthermore, in some studies, correlations across speech conditions were more limited (Bent et al., 2016; McLaughlin et al., 2018). Bent et al. (2016) studied intelligibility under different types of signal adversity and showed that English-speaking listeners who were good at understanding non-native (Spanish) accent were also good at understanding a regional dialect (Irish English) and disordered speech (ataxic dysarthria). These results indicated that, rather than possessing a general speech skill, listeners may possess specific cue sensitivities and/or favor perceptual strategies that allow them to be successful with particular types of listening adversity. Therefore, at present, it is hard to determine whether differences between different speech conditions stem from differences in the requirements they pose on underlying auditory mechanisms, from differences in listening effort or from methodological issues. For example, in the current study and with similar tasks, recognition of fast speech was poorer than that of speech in babble, but using different fast talkers or a more challenging SNR could have changed this pattern. Further studies with conditions matched for accuracy might shed further light on this issue if listening effort is tracked and compared across conditions. As for older adults with hearing impairment, both general speech skills and specific cue sensitivities/perceptual strategies decline with aging. Further research is needed to understand individual differences in those declines, which could help shed light on the varying degrees of benefit from current rehabilitative strategies.

In contrast to previous work in older adults (e.g., Salthouse, 1994, 1996; Pichora-Fuller et al., 1995; Humes, 2007; Rotman et al., 2020b), in the present study, cognitive abilities (working memory, vocabulary, and selective attention) were not significant predictors of performance on any of the speech perception tasks. This suggests that the relationship between cognition and speech perception is not straightforward. Indeed, Akeroyd (2008) found inconsistencies across studies both when the speech and the cognitive tasks varied across studies, and also when the assessed cognitive domain (e.g., working memory) was constant and only the speech task differed. However, task and stimulus related factors do not provide a sufficient account for the discrepancies across studies, because in the current study, we used the same time-compressed, fast speech and cognitive tasks as in a previous study from our lab in which we did find an association between fast speech recognition and vocabulary (Rotman et al., 2020b). A recent review by Dryden et al. (2017) highlighted that not only do measures of speech in noise perception and cognitive tasks vary greatly across published studies, but research participant samples vary widely as well and can include any combination of young and old listeners with or without hearing loss, tested under aided or unaided listening conditions. Consistent with this view, in the current study, effect sizes (expressed in odd ratios) were similar to those observed in our previous study. Furthermore, based on our previous data (Rotman et al., 2020b), statistical power was adequate. On the other hand, hearing levels were more variable and this increased variability may have contributed to the lack of significant effects.

The current finding that hearing aid use had no effect on degraded speech perception is consistent with that of Rotman et al. (2020b). However, this finding contradicts previous research showing improved speech perception following hearing aid use (Gatehouse, 1992; Munro and Lutman, 2003; Lavie et al., 2015; Habicht et al., 2016; Dawes and Munro, 2017; Wright and Gagné, 2020). One potential explanation for this could be that in our study, the average hearing loss (PTA) in hearing aid users was approximately 10 dB more severe than in non-users (see Table 1). This greater severity of hearing loss could have masked a hearing aid induced effect despite the inclusion of PTAs in statistical modeling. Methodological differences, including: timing and duration of hearing aid use (e.g., Gatehouse, 1992; Munro and Lutman, 2003), variability of outcome measures (e.g., Larson et al., 2000; Humes et al., 2001), and lack of baseline tests before starting to use the hearing aids (e.g., Vogelzang et al., 2021), can also account for the discrepancy between studies. The above differences highlight the need for further research on speech processing among hearing aid users. For example, future studies should include an unaided condition for the group with hearing aids and an aided condition for the group without hearing aids. This could test differences between the effects of hearing aid use and the effects of amplification during testing, without using hearing aids between test sessions.

## Data Availability Statement

The data from this study is available at Open Science Foundation; https://osf.io/sreq4.

## Ethics Statement

This study involves human participants. It was reviewed and approved by the ethics committee of the Faculty of Social Welfare and Health Sciences, University of Haifa. Protocol 362/18 participants provided their written informed consent to participate in this study.

## Author Contributions

LL and KB designed the study, prepared the study materials, and edited the manuscript. LS recruited study participants, collected and analyzed the data, and wrote the manuscript with oversight and conceptual guidance from KB and LL. All authors approved the submitted version.

## Funding

This study was supported by the Israel Science Foundation (grant number 206/18).

## Conflict of Interest

The authors declare that the research was conducted in the absence of any commercial or financial relationships that could be construed as a potential conflict of interest.

## Publisher’s Note

All claims expressed in this article are solely those of the authors and do not necessarily represent those of their affiliated organizations, or those of the publisher, the editors and the reviewers. Any product that may be evaluated in this article, or claim that may be made by its manufacturer, is not guaranteed or endorsed by the publisher.
